# Establishment of a circular RNA regulatory stemness-related gene pair signature for predicting prognosis and therapeutic response in colorectal cancer

**DOI:** 10.3389/fimmu.2022.934124

**Published:** 2022-07-25

**Authors:** Qian Chen, Peng Tang, Huishen Huang, Xiaoqiang Qiu

**Affiliations:** ^1^ Department of Epidemiology, School of Public Health, Guangxi Medical University, Nanning, China; ^2^ Department of Experimental Research, Guangxi Medical University Cancer Hospital, Nanning, China

**Keywords:** colorectal cancer, circRNA, stemness-related gene pair signature, immune, nomogram

## Abstract

**Background:**

Colorectal cancer (CRC) is a common malignant tumor of the digestive tract with a poor prognosis. Cancer stem cells (CSCs) affect disease outcomes and treatment responses in CRC. We developed a circular RNA (circRNA) regulatory stemness-related gene pair (CRSRGP) signature to predict CRC patient prognosis and treatment effects.

**Methods:**

The circRNA, miRNA, and mRNA expression profiles and clinical information of CRC patients were obtained from The Cancer Genome Atlas (TCGA) and Gene Expression Omnibus (GEO) databases. CRSRGPs were established based on stemness-related genes in the competing endogenous RNA (ceRNA) network. A CRSRGP signature was generated using the least absolute shrinkage and selection operator (Lasso) and Cox regression analysis of TCGA training set. The prognosis was predicted by generating a nomogram integrating the CRSRGP signature and clinicopathologic features. The model was validated in an external validation set (GSE17536). The antitumor drug sensitivity and immunotherapy responses of CRC patients in the high-risk group (HRG) and low-risk group (LRG) were evaluated by the pRRophetic algorithm and immune checkpoint analysis.

**Results:**

We established an 18-CRSRGP signature to predict the prognosis and treatment responses of CRC patients. In the training and external validation sets, risk scores were used to categorize CRC patients into the HRG and LRG. The Kaplan–Meier analysis showed a poor prognosis for patients in the HRG and that subgroups with different clinical characteristics had significantly different prognoses. A multivariate Cox analysis revealed that the CRSRGP signature was an independent prognostic factor. The nomogram integrating clinical features and the CRSRGP signature efficiently predicted CRC patient prognosis, outperformed the current TNM staging system, and had improved practical clinical value. Anticancer drug sensitivity predictions revealed that the tumors of patients in the HRG were more sensitive to pazopanib, sunitinib, gemcitabine, lapatinib, and cyclopamine. Analysis of immune checkpoint markers demonstrated that patients in the HRG were more likely to benefit from immunotherapy.

**Conclusion:**

An efficient, reliable tool for evaluating CRC patient prognosis and treatment response was established based on the 18-CRSRGP signature and nomogram.

## Introduction

Colorectal cancer (CRC) has become an increasingly serious threat to human health and has the third-highest incidence rate and second-highest mortality rate of all cancers (1). Despite recent improvements in diagnosis and treatment, such as the widespread use of colonoscopy and surgery, the overall survival rate for CRC, especially metastatic CRC, remains low at 14% (2). To evaluate tumor prognosis, the TNM staging system has been defined as the gold standard. However, patients in the same TNM stage have different treatment responses and prognoses (3). Hence, it is crucial to discover new markers to evaluate CRC patient prognosis and treatment response.

Circular RNA (circRNA) is a non-coding RNA in which the loop structure is closed, and it provides a new candidate for tumor diagnosis and treatment (4, 5). The expression of circRNAs in a variety of tumors has been reported to be abnormal, which affects tumorigenesis and tumor development. A recent study showed that circIL4R is at high levels in the serum of CRC patients and is positively correlated with poor prognosis in CRC. CircIL4R competes with miR-761 to upregulate TRIM29 expression and promote CRC progression (6). In gastric cancer, high expression levels of circDLG1 were correlated with poor prognosis in immunotherapy-treated patients and promoted the migration, invasion, and immune escape of gastric cancer cells (7). Cancer stem cells (CSCs) are considered to underlie tumorigenesis, metastasis, and recurrence and have the characteristics of self-renewal and differentiation potential (8). The presence of CSCs in CRC affects malignant progression and treatment sensitivity (9, 10). CircRNAs were found to affect cancer stemness. Hsa_circ_0026628 levels were reported to be upregulated in CRC cells and to promote the epithelial–mesenchymal transition (EMT) and stemness of CRC cells by regulating miR346/SP1 (11). Hsa_circ_001680 levels are upregulated in CRC tissues and promote the stemness and drug resistance of CRC cells *via* the miR-340/BMI1 axis (12). In addition, circRNAs were found to be prognostic markers for cancer patients. In a recent study, researchers established a signature based on a circRNA-associated competing endogenous RNA (ceRNA) network to predict the prognosis of lung cancer patients (13). In prostate cancer, autophagy-related circRNAs were used to divide patients into two groups, and patients in the high-risk group (HRG) were reported to be more likely to develop a biochemical recurrence (14). However, prognostic markers based on circRNA regulatory stemness-related gene pairs (CRSRGPs) have not been studied in CRC.

The main goal of this study was to establish a CRSRGP signature and nomogram to improve the ability to assess the prognosis and treatment response of CRC patients. We established CRSRGPs by integrating the expression profiles and clinical data of CRC patients from The Cancer Genome Atlas (TCGA) and Gene Expression Omnibus (GEO) databases. Then, a CRSRGP signature was developed through the least absolute shrinkage and selection operator (Lasso) Cox regression to evaluate the prognosis and treatment response of CRC patients. This signature was combined with clinical information to generate a nomogram to predict the prognosis of individual CRC patients, and the accuracy of the model was verified with an external validation set. Our findings will provide new strategies for predicting prognosis and clinical treatment outcomes for CRC.

## Methods

### Data download and processing

We downloaded RNA-seq (473 CRC tissues and 41 adjacent tissues) and miRNA-seq data (457 CRC tissues and 8 adjacent tissues) and clinical data from TCGA database (15). Clinical data included age, sex, stage, TNM classification, the presence of colon polyps, survival time, and outcome. The following criteria were used for exclusion: 1) the histological diagnosis was not standard, 2) the specimens did not have complete clinical data available, and 3) the clinical follow-up time was less than 30 days. A total of 416 patients were included after applying the exclusion criteria. The GSE17536 dataset containing gene expression data and clinical data for CRC patients was obtained from the GEO database as an external verification set, and 169 patients were included after applying the same exclusion criteria (16). CircRNA expression data were obtained from GSE138589 (6 CRC tissues and 6 adjacent tissues) and GSE126094 (10 CRC tissues and 10 adjacent tissues) in the GEO database (16). CRC stemness-related genes were obtained from GSE24747 (3 CD133+ sorted Caco-2 cells and 3 CD133− sorted Caco-2 cells) (16). Regarding data processing, for probes that were mapped to the same gene multiple times, the median was used to represent its expression level. Probes corresponding to multiple genes were deleted. Data points with no expression or a mean value of less than 0.5 were removed. We used the “limma” and “SVA” packages for batch normalization in R.

### Identification of differentially expressed genes

We used the “limma” package in R to identify differentially expressed circRNAs (DEcircRNAs). Differentially expressed miRNAs (DEmiRNAs) and differentially expressed mRNAs (DEmRNAs) were analyzed using the “edgeR” package in R. The cutoff values for differentially expressed genes (DEGs) were set to |log2-fold change (FC)| > 1.0 and p < 0.05. The cutoff values for stemness-related mRNAs were set to |log2FC| > 0.58 and p < 0.05.

### Constructing the stemness-related competing endogenous RNA network

We predicted the target miRNAs of the DEcircRNAs through the Cancer-Specific CircRNA Database (CSCD), and these target miRNAs were further screened within the DEmiRNAs to obtain targeted DEmiRNAs (17). Next, we predicted the mRNAs targeted by the identified DEmiRNAs using the miRDB and TargetScan databases and obtained targeted stemness-related DEmRNAs based on the intersection of targeted mRNAs, DEmRNAs, and stemness-related mRNAs (18, 19). Finally, the stemness-related ceRNA network consisting of circRNA–miRNA and miRNA–mRNA pairs were visualized by Cytoscape (20).

### Construction and verification of the circular RNA regulatory stemness-related gene pair signature

To avoid measurement errors between different samples, the expression levels of circRNA regulatory stemness-related genes were compared pairwise to obtain a score for each CRSRGP following a previously described method (21). A CRSRGP score of 1 was assigned if CRSRGP 1 was greater than CRSRGP 2; otherwise, the CRSRGP score was 0. In the training set, CRSRGPs were further screened by univariate Cox analysis to obtain CRSRGPs related to prognosis. Using the “glmnet” package in R, we established the CRSRGP signature by Lasso Cox regression to estimate CRC outcome (iteration = 1,000). The CRSRGP signature risk score = ∑ βCRSRGPi × ExpCRSRGPi (where β is the coefficient and Exp is the expression of the CRSRGP). The same formula was also used to calculate the risk score in the external validation analysis to verify the accuracy of the CRSRGP signature.

### Establishment of a nomogram and validation

Based on the training set, a nomogram comprising clinical features and risk score was established using the R package “rms” to predict CRC patient prognosis. The area under the receiver operating characteristic (ROC) curve (AUC) was used to assess the nomogram prediction accuracy. A calibration plot was used to assess the agreement between the probability predicted by the nomogram and the observed probability. In addition, the performance of the nomogram was verified by external validation.

### Functional enrichment analysis

To study the biological mechanism of CRSRGP signature regulation, we performed Gene Ontology (GO) and Kyoto Encyclopedia of Genes and Genomes (KEGG) analyses using the R package “clusterProfiler” and the KOBAS database (22). We downloaded the pathway dataset c2.cp.kegg.v7.1.symbols from the Molecular Signatures Database and analyzed the pathway differences between the HRG and low-risk group (LRG) using the “fgsea” R package (23).

### Analysis of immune infiltration and immune function

The R packages “ESTIMATE” and “CIBERSORT” were used to analyze the stromal score, immune score, and infiltration levels of 22 different immune cells in CRC patients (**Supplementary Material**). Single-sample gene set enrichment analysis (ssGSEA) was performed using the “GSEABase” and “GSVA” R packages to quantify the regulation of immune function by 13 immune-related pathways (24). We assessed differences in the immune microenvironment between the HRG and LRG by the Wilcoxon test.

### Drug susceptibility and immunotherapy prediction

The half-maximal inhibitory concentration (IC50) was calculated by the “pRRophetic” R package to evaluate the sensitivity of tumors from CRC patients in the HRG and LRG to six anticancer drugs (gefitinib, pazopanib, sunitinib, gemcitabine, lapatinib, and cyclopamine) (25). The response of CRC patients in the HRG and LRG to immunotherapy was evaluated by analyzing immune checkpoint-related genes. Higher expression levels of immune checkpoint-related genes indicated that a patient was a better candidate for immunotherapy.

### Statistical analysis

We calculated survival differences between patients in the HRG and LRG using the Kaplan–Meier (K-M) method and the log-rank test. The AUC was implemented to evaluate the accuracy of the model in predicting prognosis. The relationship between clinical characteristics and risk scores was analyzed by the chi-square test and Fisher’s exact probability test. The relation of the CRSRGP signature with the prognosis of CRC patients was analyzed by univariate and multivariate Cox models. All data analyses were performed using R 3.6.2 software, and a two-sided p < 0.05 was considered to indicate statistical significance.

## Results

### Differentially expressed genes and the competing endogenous RNA network in colorectal cancer patients

**Figure 1:**
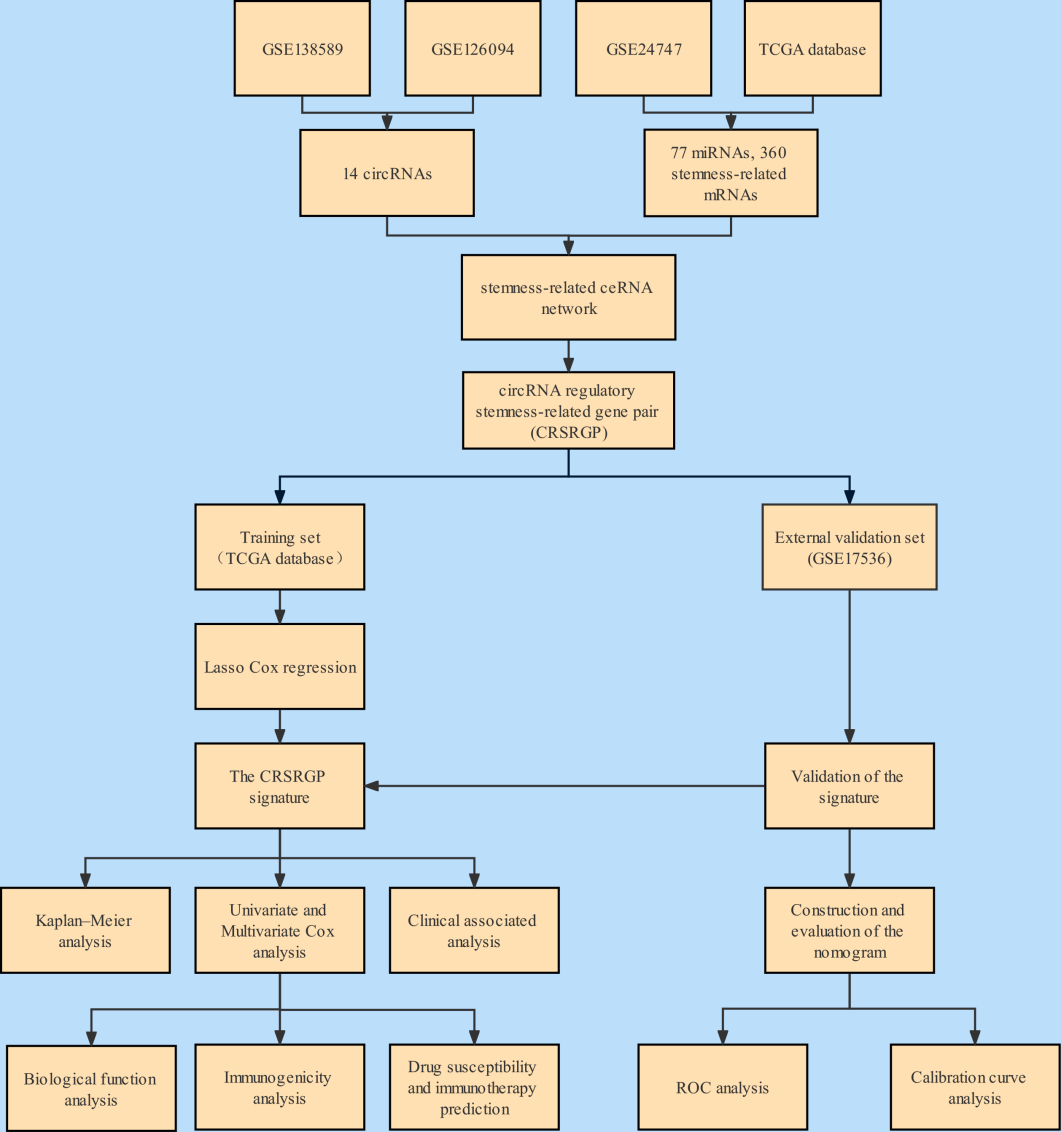
Flowchart of the analysis.

**Figure 2:**
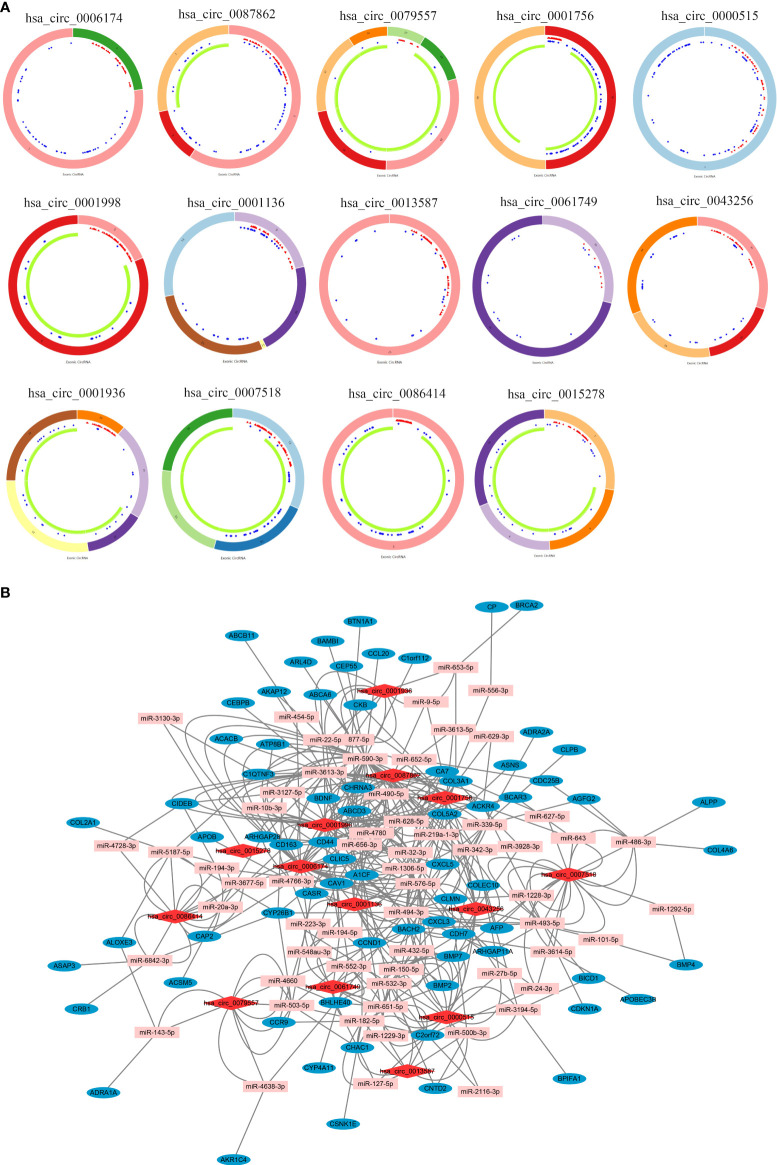
**(A)** Interaction patterns of the 14 differentially expressed circRNAs in CRC. Red, blue, and green represent microRNA response elements, RNA-binding proteins, and open reading frames, respectively. **(B)** A stemness-related network in the ceRNA network in CRC (section). Red, pink, and blue represent circRNAs, miRNAs, and mRNAs, respectively. CircRNAs, circular RNAs; CRC, colorectal cancer; ceRNA, competing endogenous RNA.

The workflow of this study is illustrated in **Figure 1**. A total of 14 DEcircRNAs in CRC tissues were obtained from the GSE138589 and GSE126094 datasets (|log2FC| >1.0 and p < 0.05) (**Figure 2A**). A total of 501 DEmiRNAs and 5325 DEmRNAs in CRC tissues were obtained from TCGA database. A total of 1,639 stemness-related mRNAs (|log2FC| > 0.58 and p < 0.05) were obtained from the GSE24747 dataset. We predicted 582 DEcircRNA target miRNAs through the CSCD and further screened the DEmiRNAs to obtain 77 targeted DEmiRNAs. Next, we predicted targeted mRNAs using the miRDB and TargetScan databases and obtained 360 targeted stemness-related DEmRNAs at the intersection of these targeted mRNAs, DEmRNAs, and stemness-related mRNAs. Finally, we built a stemness-related ceRNA network (**Figure 2B**).

### Construction and verification of the circular RNA regulatory stemness-related gene pair signature

**Figure 3:**
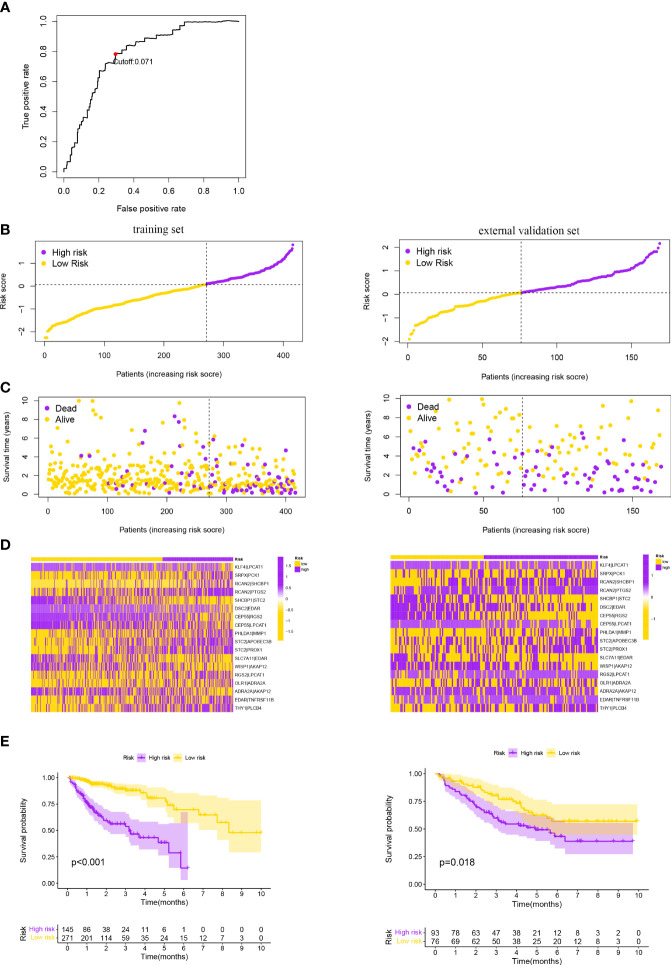
**(A)** Time-dependent ROC curve for the CRSRGP signature risk score in the training set. A risk score of 0.071 was used as the cutoff value to divide patients into the HRG and LRG. Risk score of the CRSRGP signature in the two sets. **(B)** Distribution of patients with different risk scores in the training set and external validation set. The purple and yellow points represent patients in the HRG and patients in the LRG, respectively. **(C)** Survival status of patients with different risk scores in the training set and external validation set. The purple and yellow points represent patients who were dead and alive, respectively. **(D)** Heatmap of the prognostic signature scores in the training set and external validation set. The purple and yellow points represent patients in the HRG and patients in the LRG, respectively. **(E)** Survival analysis of patients in the training set and external validation set. The survival curve shows that patients in the HRG had a poorer outcome than patients in the LRG. ROC, receiver operating characteristic; CRSRGP, circular RNA regulatory stemness-related gene pair; HRG, high-risk group; LRG, low-risk group.

Data from TCGA database were used as the training set, which included a total of 360 circRNA regulatory stemness-related genes and selected genes with a median absolute deviation >0.5 to obtain 98 circRNA regulatory stemness-related genes. After CRSRGPs with limited variation (0 or 1, <20%) were removed, 1,108 CRSRGPs were obtained. The CRSRGPs were further screened by univariate Cox analysis, and 22 CRSRGPs related to prognosis were obtained (p < 0.018). We used Lasso Cox regression to construct a signature of 18 CRSRGPs, including 24 circRNA regulatory stemness-related genes, to predict CRC patient prognosis (**Table 1**). Time-dependent ROC curve analysis determined that the best cutoff value for the signature was 0.071, and the patients were divided into the HRG and LRG according to this value (**Figures 3A–D**). The K-M analysis and log-rank test showed that patients in the HRG had lower 5-year survival rates (p < 0.001) (**Figure 3E**). To determine whether the 18-CRSRGP signature has a similar prognostic value in other populations, we selected GSE17536 as an independent external verification set. The risk score was calculated using the same algorithm (**Figures 3B–D**). We found that patients in the HRG had a poorer prognosis than patients in the LRG (p = 0.018) (**Figure 3E**). The results were consistent with those of the training set.

**Table 1:** List of the 18 CRSRGPs in the prognostic signature.

Gene pair 1	Full name	Gene pair 2	Full name	Coefficient
KLF4	Krüppel-like factor 4	LPCAT1	Lysophosphatidylcholine acyltransferase 1	−0.388
SRPX	Sushi repeat-containing protein X-linked	PCK1	Phosphoenolpyruvate carboxykinase 1	0.068
RCAN2	Regulator of calcineurin 2	SHCBP1	SHC binding and spindle associated 1	0.298
RCAN2	Regulator of calcineurin 2	PTGS2	Prostaglandin-endoperoxide synthase 2	0.236
SHCBP1	SHC binding and spindle associated 1	STC2	Stanniocalcin 2	−0.308
DSC2	Desmocollin 2	EDAR	Ectodysplasin A receptor	−0.252
CEP55	Centrosomal protein 55	RGS2	Regulator of G protein signaling 2	−0.302
CEP55	Centrosomal protein 55	LPCAT1	Lysophosphatidylcholine acyltransferase 1	−0.348
PHLDA1	Pleckstrin homology like domain family A member 1	MMP1	Matrix metallopeptidase 1	0.471
STC2	Stanniocalcin 2	APOBEC3B	Apolipoprotein B mRNA editing enzyme catalytic subunit 3B	0.135
STC2	Stanniocalcin 2	PROX1	Prospero homeobox 1	0.528
SLC7A11	Solute carrier family 7 member 11	EDAR	Ectodysplasin A receptor	−0.312
WISP1	Cellular communication network factor 4	AKAP12	A-kinase anchoring protein 12	−0.285
RGS2	Regulator of G protein signaling 2	LPCAT1	Lysophosphatidylcholine acyltransferase 1	0.289
OLR1	Oxidized low density lipoprotein receptor 1	ADRA2A	Adrenoceptor alpha 2A	0.266
ADRA2A	Adrenoceptor alpha 2A	AKAP12	A-kinase anchoring protein 12	−0.065
EDAR	Ectodysplasin A receptor	TNFRSF11B	TNF receptor superfamily member 11b	0.134
THY1	Thy-1 cell surface antigen	PLCB4	Phospholipase C beta 4	0.389

CRSRGPs, circular RNA regulatory stemness-related gene pairs.

### Subgroup analysis and Cox analyses of the circular RNA regulatory stemness-related gene pair signature

**Figure 4:**
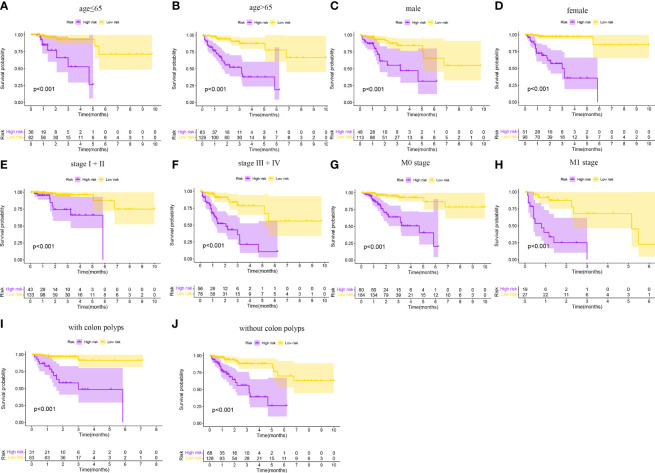
Subgroup analyses of the overall survival of CRC patients in TCGA database. **(A)** Age ≤ 65 years. **(B)** Age > 65 years. **(C)** Male. **(D)** Female. **(E)** Stage I+II. **(F)** Stage III+IV. **(G)** M0 stage. **(H)** M1 stage. **(I)** With colon polyps. **(J)** Without colon polyps. CRC, colorectal cancer; TCGA, The Cancer Genome Atlas.

**Figure 5:**
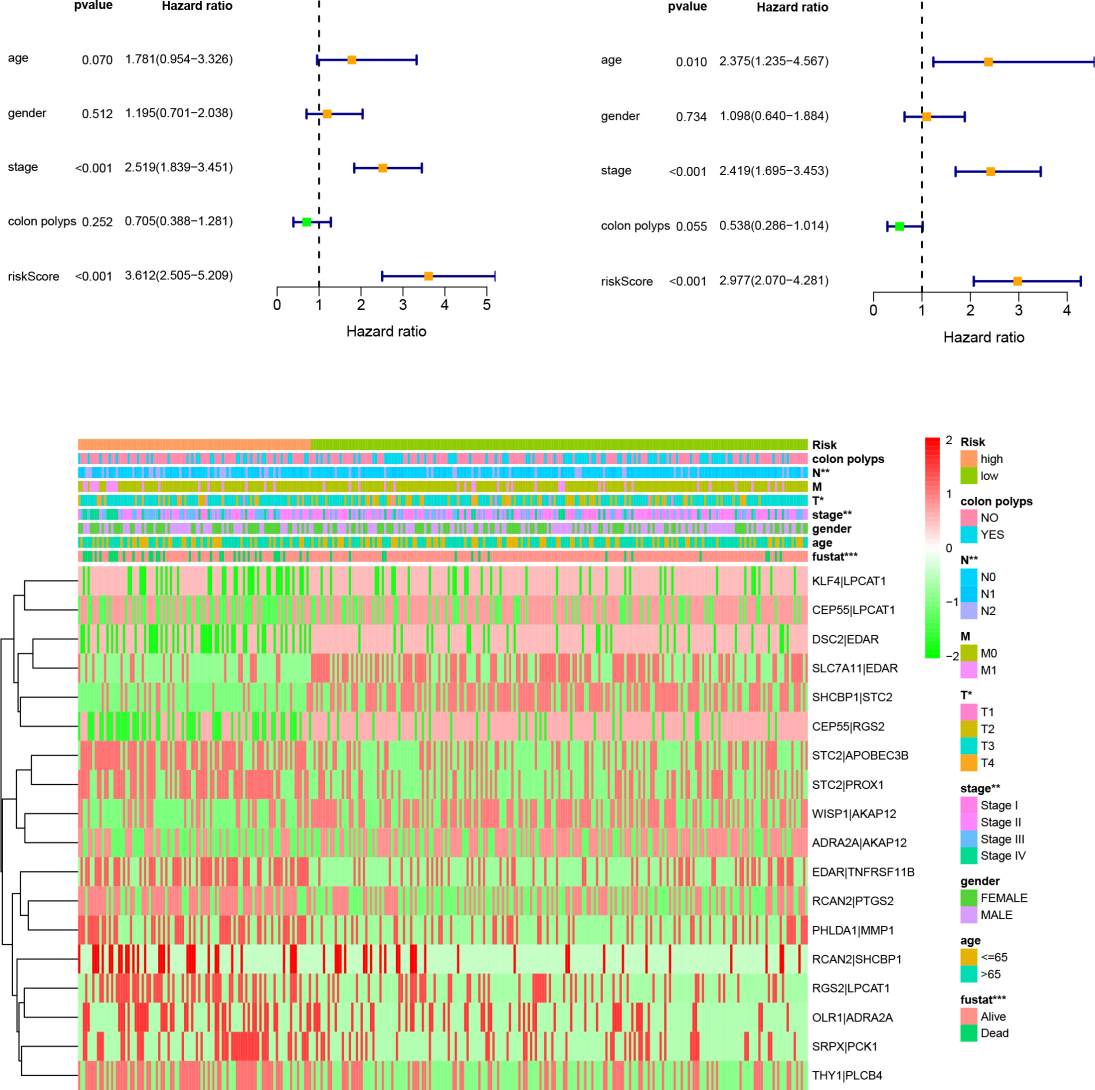
Univariate and multivariate Cox analyses of CRC. **(A)** Univariate analysis. **(B)** Multivariate analysis. **(C)** Relationship between the CRSRGP signature and clinical characteristics (***p < 0.001, **p < 0.01, *p < 0.05). CRC, colorectal cancer; CRSRGP, circular RNA regulatory stemness-related gene pair.

As shown in **Figure 4**, the CRSRGP signature can function as a prognostic indicator in subgroups of patients with different clinical characteristics in TCGA database. In the age (≤65 and >65 years), sex (male and female), clinical stage (I+II and III+IV), M stage (M0 and M1), and colon polyps (yes and no) groups, the overall survival was worse in the HRG than in the LRG (p < 0.05). Next, we investigated whether the CRSRGP signature is an independent prognostic risk factor for CRC patients by performing univariate and multivariate Cox analyses. Univariate Cox analysis showed that stage and risk scores had prognostic significance (**Figure 5A**). Multivariate Cox analysis showed that age (hazard ratio (HR) = 2.375, 95% CI = 1.235~4.567, p = 0.010), stage (HR = 2.419, 95% CI = 1.695~3.453, p < 0.001), and risk score (HR = 2.977, 95% CI = 2.070~4.281, p < 0.001) were independent risk factors for CRC prognosis (**Figure 5B**). The result revealed that the risk score of the CRSRGP signature was different at different T stages (p = 0.01), N stages (p = 0.002), and all stages (p = 0.005) (**Figure 5C**).

### Establishment and evaluation of the nomogram for predicting prognosis

**Figure 6:**
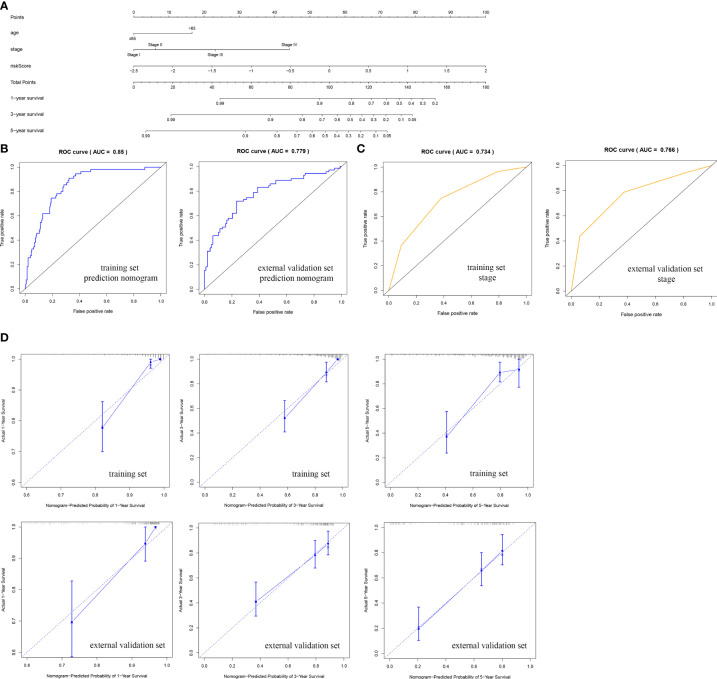
Nomogram used to predict the prognosis of CRC patients at 1, 3, and 5 years. **(A)** Nomogram based on the CRSRGP signature and clinical features. ROC analysis of the ability to predict overall survival based on **(B)** the nomogram and **(C)** stage. **(D)** Calibration curve for the predictive accuracy of the nomogram. CRC, colorectal cancer; CRSRGP, circular RNA regulatory stemness-related gene pair; ROC, receiver operating characteristic.

We established a nomogram to assess the survival of CRC patients. The corresponding scores for each factor (age, stage, and risk) can be summed to obtain the patient’s total score, with higher patient scores indicating a worse prognosis (**Figure 6A**). We analyzed the accuracy of the nomogram for CRC prognosis by ROC analysis, and the AUC of the predictive nomogram in the training set was 0.850, which was higher than that for stage (0.734) (**Figures 6B, C**). As shown by the calibration curve, the nomogram showed a good fit between the predicted and actual prognostic observations at 1, 3, and 5 years (**Figure 6D**). In addition, the accuracy of the nomogram was verified in the external validation set. The AUC of the predictive nomogram was 0.779, which was higher than that for stage (0.766) (**Figures 6B, C**). The calibration curve also showed good accuracy, which was consistent with the findings in the training set (**Figure 6D**).

### Biological function analysis of the circular RNA regulatory stemness-related gene pair signature

**Figure 7:**
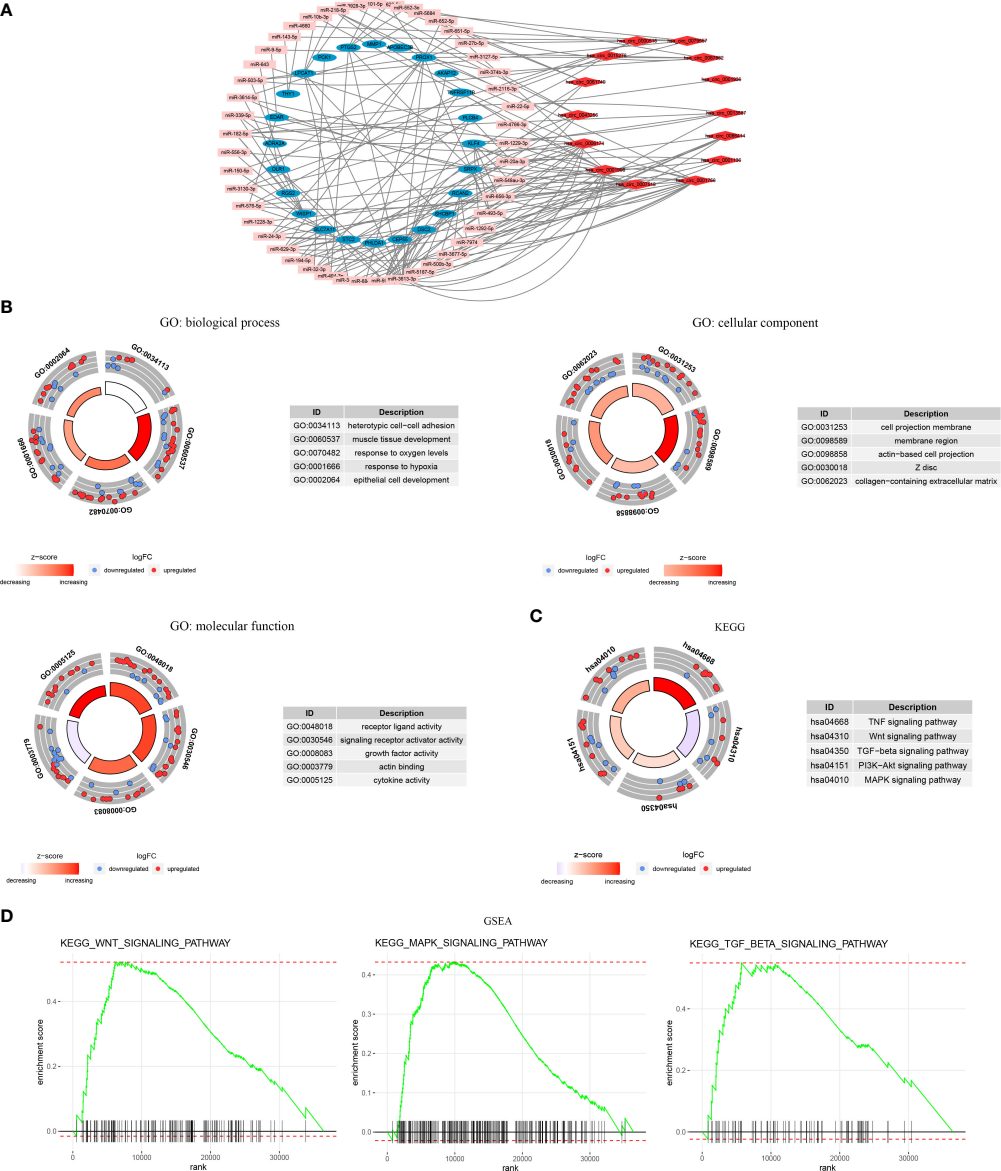
Functional enrichment analysis of CRSRGPs. **(A)** CRSRGP ceRNA network. **(B)** GO enrichment analysis. **(C)** KEGG enrichment analysis. **(D)** Gene set enrichment analysis. CRSRGPs, circular RNA regulatory stemness-related gene pairs; ceRNA, competing endogenous RNA; GO, Gene Ontology; KEGG, Kyoto Encyclopedia of Genes and Genomes.

The CRSRGP ceRNA network is shown in **Figure 7A**. We performed GO and KEGG analyses on circRNA regulatory stemness-related genes to clarify their biological functions. The biological process (BP) terms included heterotypic cell−cell adhesion and epithelial cell development, the cellular component (CC) terms included the membrane region and collagen-containing extracellular matrix, and the molecular function (MF) terms included growth factor activity and actin binding (**Figure 7B**). KEGG analysis indicated that the signature genes were enriched for the MAPK, WNT, PI3K–AKT, and TNF signaling pathways (**Figure 7C**). In addition, GSEA results showed enrichment for pathways related to tumor progressions, such as the WNT, MAPK, and TGF-β signaling pathways (**Figure 7D**).

### Relationship between the circular RNA regulatory stemness-related gene pair signature and immunogenicity

**Figure 8:**
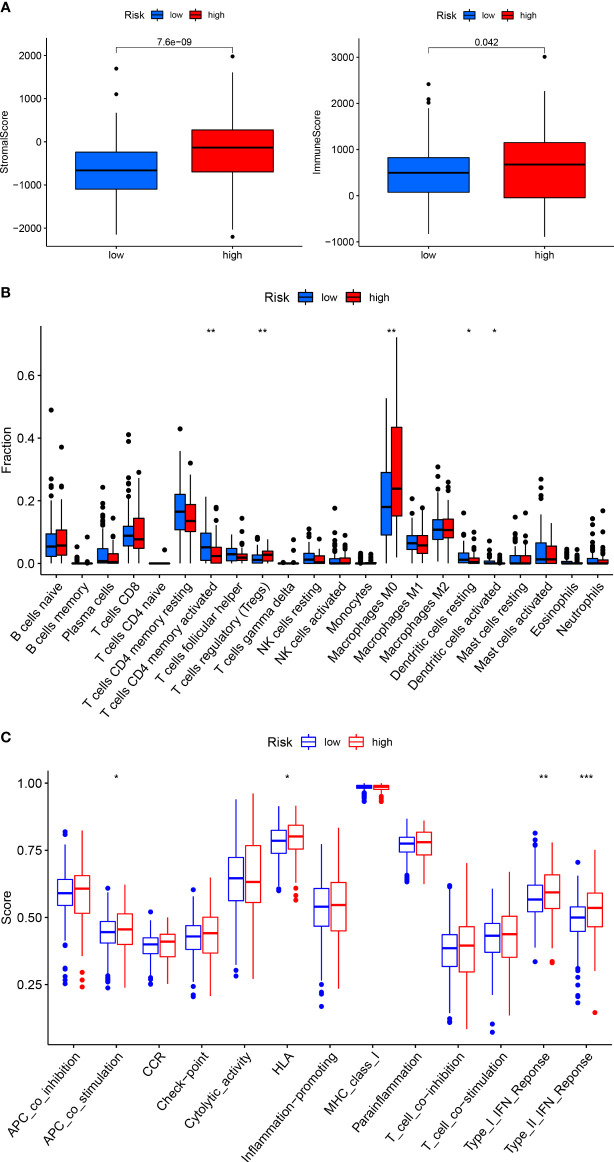
Correlation between the CRSRGP signature and immunogenicity. The relationship between the CRSRGP signature and **(A)** the tumor microenvironment, **(B)** immune cell infiltration, and **(C)** immune function (***p < 0.001, **p < 0.01, *p < 0.05). CRSRGP, circular RNA regulatory stemness-related gene pair.

We used ESTIMATE and CIBERSORT to assess the microenvironment and immune cell infiltration of tumors from CRC patients in the HRG and LRG. The results showed that patients in the HRG had higher stromal scores and immune scores in the tumor microenvironment than those LRG patients (**Figure 8A**). There was considerable infiltration of M0 macrophages and regulatory T cells (Tregs) in the immune microenvironment of patients in the HRG, indicating different immune states in patients in the HRG and LRG (**Figure 8B**). Then, we used the ssGSEA algorithm to evaluate the immune function and found that Type_I_IFN_Response and Type_II_IFN_Response were activated in patients in the HRG, indicating that patients in the HRG were in a state of immunosuppression and should receive immunotherapy (**Figure 8C**).

### Drug susceptibility and immunotherapy prediction

**Figure 9:**
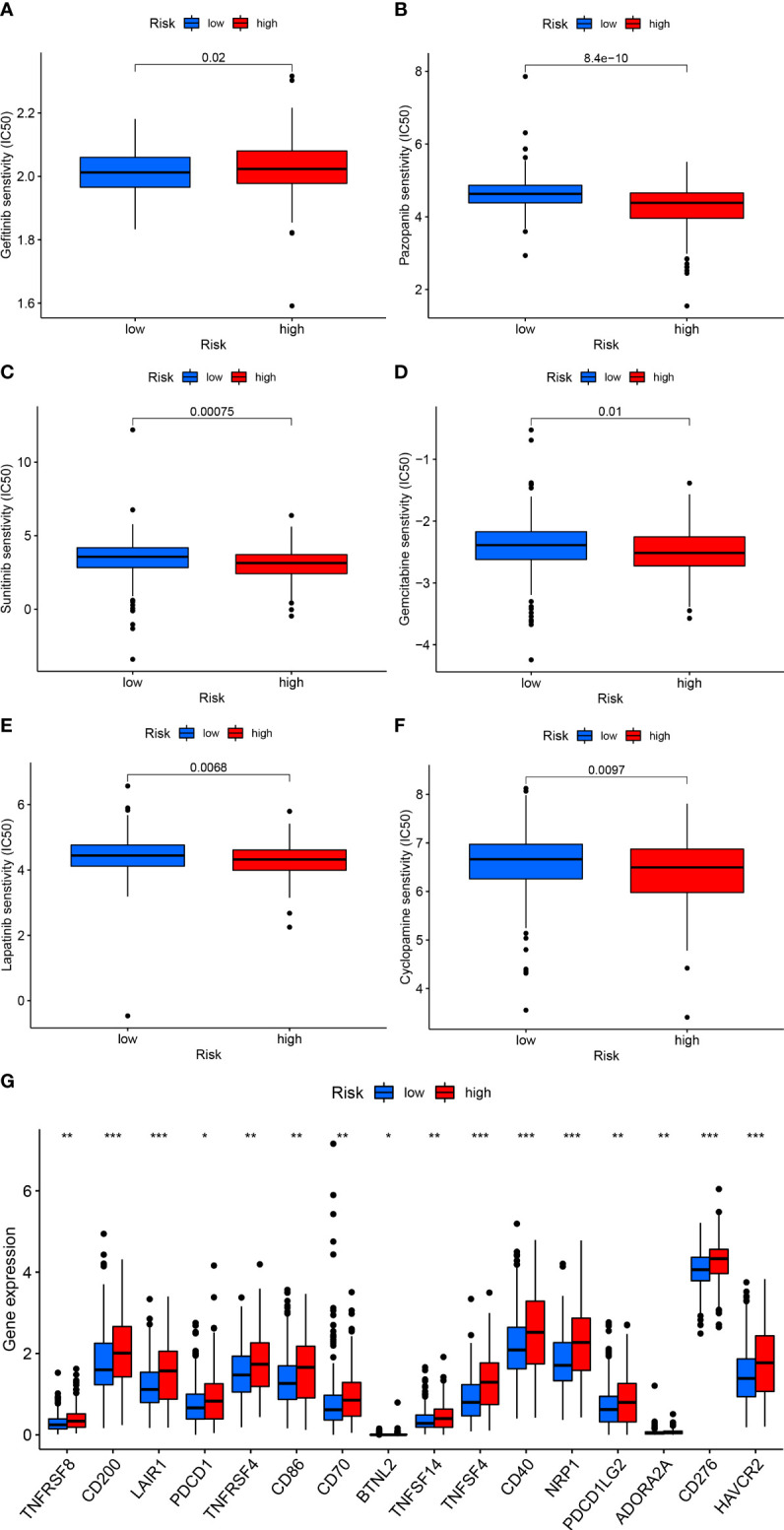
CRSRGP signature in CRC treatment. Differences in the estimated IC50 values of **(A)** gefitinib, **(B)** pazopanib, **(C)** sunitinib, **(D)** gemcitabine, **(E)** lapatinib, and **(F)** cyclopamine between the HRG and LRG. **(G)** Differences in the expression levels of immune checkpoint proteins between the HRG and LRG (***p < 0.001, **p < 0.01, *p < 0.05). CRSRGP, circular RNA regulatory stemness-related gene pair; CRC, colorectal cancer; IC50, half-maximal inhibitory concentration; HRG, high-risk group; LRG, low-risk group.

There is increasing evidence that cancer stemness is associated with resistance to immunotherapy and anticancer drug therapy, so we investigated the value of the CRSRGP signature in predicting CRC treatment outcomes. The IC50 values ​​of six anticancer drugs (gefitinib, pazopanib, sunitinib, gemcitabine, lapatinib, and cyclopamine) were calculated by the pRRophetic algorithm to predict the responses of patients in the HRG and LRG to anticancer drug treatment. The results showed that patients in the HRG may benefit from treatment with pazopanib, sunitinib, gemcitabine, lapatinib, and cyclopamine, while patients in the LRG may benefit from treatment with gefitinib (**Figures 9A–F**). Regarding immunotherapy, we compared the sensitivity of the HRG and LRG to common immune checkpoint inhibitors. In the HRG, the expression levels of PDCD1 (PD1), PDCD1LG2 (PDL2), and CD276 were higher than those in the LRG, suggesting that patients in the HRG were more sensitive to immune checkpoint inhibitors (**Figure 9G**). The above results suggest that the CRSRGP signature can be used to predict sensitivity to anticancer drug treatment and immunotherapy in the future.

## Discussion

In recent years, CRC has become an increasingly serious threat to human health globally and has brought a serious burden to society (1). Despite breakthroughs in surgery, radiotherapy, chemotherapy, and immunotherapy, CRC is still prone to metastasize and has a poor survival rate. CSCs exist in CRC, wherein they affect recurrence, metastasis, and treatment outcomes. CircRNA-based signatures can accurately predict the prognosis of cancer patients, and this approach has attracted much attention (13, 14). However, it is unknown whether CRC prognosis can be predicted based on the CRSRGP signature.

Our research focused on the CRSRGP signature. We first established a stemness-related ceRNA network for CRC based on RNA expression profile data in TCGA and GEO databases. We established CRSRGPs from circRNA regulatory stemness-related genes in the ceRNA network. In TCGA training set, we identified 22 CRSRGPs associated with prognosis. A prognostic model of the 18-CRSRGP signature was generated using Lasso Cox regression. Survival analysis showed a poorer prognosis for CRC patients in the HRG than in LRG patients, a finding that was consistent with that obtained when assessed in the GSE17536 cohort. Multivariate Cox analysis showed that the CRSRGP signature was an independent risk factor for the prognosis of CRC patients. We established a nomogram to predict prognosis in CRC patients based on clinical information and risk scores. The results show that the model is more accurate than the TNM staging system. Consistent results were obtained in the external validation set. Pathway enrichment analysis indicated that the CRSRGP signature was enriched for the WNT and MAPK signaling pathways and other tumor-related pathways. In addition, the CRSRGP signature may help guide CRC clinical treatment. Regarding immunotherapy, patients in the HRG were better candidates for immunotherapy than those in the LRG. Regarding the prediction of drug sensitivity, the IC50 values of six anticancer drugs were estimated to predict the responses of patients in the HRG and LRG to anticancer drug treatment. Patients in the HRG may benefit from therapy with pazopanib, sunitinib, gemcitabine, lapatinib, and cyclopamine, while patients in the LRG may benefit from treatment with gefitinib, indicating that the CRSRGP signature can be used for the personalized treatment of CRC patients.

One of the components of our established stemness-related ceRNA network for CRC, circ_0006174, was identified as upregulated in CRC tissues and cells, and its high expression was associated with larger tumor volume and advanced stage (26). Wei et al. performed cell function experiments and xenograft tumor model analyses to demonstrate that circ_0006174 can promote CRC cell proliferation *in vitro* and *in vivo* (27). Another study showed that circ_0006174 can enhance chemoresistance in CRC through the miR-1205/CCND2 axis (28). Huang et al. found that circ_0087862 can promote the malignant behavior of CRC by regulating miR-142-3p/BACH1 (29). Fang et al. demonstrated that silencing circ_0001136 inhibited the colony formation and invasion abilities of CRC cells, which is expected to become a new therapeutic target (30). However, there have been few studies on other circRNAs in CRC, and further study is warranted. Regarding the key circRNA regulatory stemness-related genes in the network, SLC7A11 levels were upregulated in CRC stem cells, and the viability of CRC stem cells was reduced after SLC7A11 expression was inhibited (31). Another stemness gene, PROX1, has been demonstrated to promote the proliferation of CRC stem cells and malignant tumor progression processes (32). MMP1 can promote the development of CRC through EMT and the AKT signaling pathway (33). Zhang et al. demonstrated with multicenter data that CRC patients with high STC2 expression are prone to developing distant metastasis; STC2 can be used as a prognostic marker (34). OLR1 can promote CRC cell chemoresistance by upregulating c-MYC and SULT2B1 levels (35). Yu et al. demonstrated that KLF4 overexpression leads to an increase in CSC population and promotes the invasion of breast cancer cells (36). KLF4 can promote the abilities of invasion and self-renewal of CSCs, which may serve as a therapeutic target for brain metastasis of breast cancer (37). Zhang et al. demonstrated that NANOG mediates tobacco smoke-induced enhancement of renal CSC properties (38). Regarding regulatory function, previous literature reported that the WNT signaling pathway is the main pathway that promotes CRC stemness (39). Tang et al. revealed that TM4SF1 is a key gene in CRC recurrence and metastasis and promotes CRC stemness through the WNT signaling pathway (40). AGR3 can activate the WNT signaling pathway and upregulate the expression of stemness-related genes to enhance the stemness of CRC cells. Thus, AGR3 is expected to become a new therapeutic target (41). Wang et al. found that cholesterol can enhance the stemness of CRC cells through activation of the MAPK signaling pathway (42). The above reports are consistent with our findings; however, the regulatory relationship with the stemness-related ceRNA network remains unclear. To identify new strategies for the individualized treatment of CRC, it is necessary to conduct experimental research on the regulatory mechanism of the stemness-related ceRNA network.

In recent years, nomograms have been widely used as individualized and accurate evaluation tools to evaluate cancer prognosis. Zhang et al. established a nomogram based on a ferroptosis-related lncRNA signature, age, stage, and T stage, which showed good clinical application value (43). Researchers established a ceRNA nomogram composing 13 genes that can individually assess the outcome of CRC patients (44). Furthermore, nomograms have been established using invasion-related gene signature, tumor mutation burden, immune-related gene signature, and clinical characteristics for the individualized prognostic assessment of CRC patients (45, 46). In this study, the nomogram based on clinical features and the CRSRGP signature has higher accuracy than recently reported nomograms (**Table 2**).

**Table 2:** Comparison of studies on existing nomograms for CRC.

Databases	Symbols	Survival event	AUC	Reference
TCGA	Ferroptosis-related lncRNA signature, age, stage, and T stage	OS	0.736	Zhang et al., 2021 [43]
TCGA	CeRNA network genes	OS	0.693	Chang et al., 2020 [44]
TCGA	Invasion-related gene signature and M stage	OS	0.68	Dong et al., 2021 [45]
TCGA	Immune-related gene signature, tumor mutation burden, and clinicopathologic features	OS	0.737	Zhou et al., 2021 [46]
TCGA, GEO	CRSRGP signature, age, and stage	OS	0.85	Our nomogram

CRC, colorectal cancer; AUC, area under the receiver operating characteristic curve; TCGA, The Cancer Genome Atlas; ceRNA, competing endogenous RNA; GEO, Gene Expression Omnibus; OS, overall survival; CRSRGPs, circular RNA regulatory stemness-related gene pair.

Although we established our CRSRGP signature using different databases, there are still some shortcomings. First, our study sample was derived from a retrospective study, and the findings need to be validated in a multicenter prospective study with a larger sample. Second, we established a stemness-related ceRNA network through a bioinformatics approach. The stemness regulation and immunity correlated potential need to be studied *in vitro* and *in vivo*.

## Conclusions

We established a CRSRGP signature to predict the prognosis of CRC patients, and this signature can guide clinicians to make specific treatment decisions. In addition, the nomogram generated based on the CRSRGP signature has better clinical value than the TNM staging system at predicting prognosis. We expect the CRSRGP signature to provide new insights into the prognostic prediction and precise treatment of CRC patients.

## Data Availability Statement

Publicly available datasets were analyzed in this study. These data can be found here: https://cancergenome.nih.gov/ and https://www.ncbi.nlm.nih.gov/geo/. The accession number(s) can be found in the article/**supplementary material**.

## Author Contributions

XQ and QC were responsible for the overall study design. QC, PT, and HH performed the data analysis. QC drafted the paper. All authors read and approved the final manuscript.

## Funding

This work was supported by the National Natural Science Foundation of China (81960613), the Innovation Project of Guangxi Graduate Education (YCBZ2022083), the Promoting Project of Basic Capacity for Young and Middle-aged University Teachers in Guangxi (2021KY0107), and the International Communication of Guangxi Medical University Graduate Education.

## Acknowledgments

We are very grateful to Zhiyue Kang from Guangxi Medical University Cancer Hospital for their helpful support.

## Conflict of Interest

The authors declare that the research was conducted in the absence of any commercial or financial relationships that could be construed as a potential conflict of interest.

## Publisher’s Note

All claims expressed in this article are solely those of the authors and do not necessarily represent those of their affiliated organizations, or those of the publisher, the editors and the reviewers. Any product that may be evaluated in this article, or claim that may be made by its manufacturer, is not guaranteed or endorsed by the publisher.
